# Serine mistranslation induces the integrated stress response through the P stalk

**DOI:** 10.1016/j.jbc.2025.108447

**Published:** 2025-03-25

**Authors:** Hong Zhang, Jiqiang Ling

**Affiliations:** Department of Cell Biology and Molecular Genetics, The University of Maryland, College Park, Maryland, USA

**Keywords:** translational fidelity, ThrRS, AlaRS, tRNA misacylation, stress response

## Abstract

Aminoacyl-tRNA synthetases (aaRSs) are essential enzymes that support robust and accurate protein synthesis. A rapidly expanding number of studies show that mutations in aaRSs lead to multiple human diseases, including neurological disorders and cancer. How aaRS mutations impact human health is not fully understood. In particular, our knowledge of how aminoacylation errors affect stress responses and fitness in eukaryotic cells remains limited. The integrated stress response (ISR) is an adaptive mechanism in response to multiple stresses. However, chronic activation of the ISR contributes to the development of multiple diseases such as neuropathies. In this study, we show that Ser misincorporation into Ala and Thr codons, resulting from either aaRS-editing defects or mutations in tRNAs, activates the ISR. We further demonstrate that activation of the ISR by Ser mistranslation does not depend on the accumulation of uncharged tRNAs but rather requires the P stalk associated with the ribosome, implying that ribosome stalling and collision are involved. Our work highlights that certain types of aminoacylation errors can lead to chronic activation of the ISR, potentially affecting fitness and disease progression.

Aminoacyl-tRNA (aa-tRNA) synthetases (aaRSs) universally exist in all organisms and are essential enzymes required for protein synthesis. AaRSs define the first step of protein synthesis by charging cognate tRNAs with amino acids to form aa-tRNAs, which are delivered to the ribosome for nascent peptide formation (1, 2, 3). In some cases, mischarging of tRNAs by aaRSs can occur (4, 5). Most organisms have evolved aaRS-editing activities to deacylate the mischarged aa-tRNAs, and the editing function is an essential checkpoint to ensure translation fidelity (6, 7). An increasing number of studies show that mutations in aaRS genes result in various neurological diseases (*e.g.*, Charcot–Marie–Tooth [CMT] disease and microcephaly) (8, 9, 10, 11, 12), developmental delay (13, 14, 15, 16), and cancer (17, 18, 19). Other factors involved in tRNA biogenesis and tRNA modifications are also linked to multiple diseases (20, 21, 22). Revealing the physiological effects of aaRS mutations is thus critical to understanding the development of neurodegenerative diseases and searching for effective drugs to treat these diseases.

Mistranslation of the genetic code by deficient aaRSs is usually considered unfavorable for cell growth. Faithful translation of the genetic code into active protein is crucial for cell viability, as translation errors can lead to the accumulation of misfolded proteins and protein aggregates that are toxic to the cells (23, 24). It has been shown that mutations in alanyl-tRNA synthetase (AlaRS) lead to an editing defect and damage to neurons and cardiomyocytes (25). We have recently shown that mutations in the editing domain of yeast AlaRS and threonyl-tRNA synthetase (ThrRS) cause sensitivity to heat stress (26, 27). However, translational infidelity may also benefit bacteria under certain stress conditions (28, 29, 30), partially because of the activation of stress responses by moderate mistranslation.

Diverse stressful conditions activate the integrated stress response (ISR), which is an evolutionarily conserved signaling pathway that adapts cells to stresses (31). In mammalian cells, the ISR is mediated by four stress-sensing kinases (PERK, GCN2, PKR, and HRI) to reduce overall protein biosynthesis while allowing translation of specific genes to support adaptation to adverse environments (32). The ISR is also known as the general amino acid control signaling pathway in yeast (33, 34, 35). Amino acid starvation accumulates uncharged tRNAs, which bind to and activate Gcn2, a protein that structurally mimics histidyl-tRNA synthetase (36). Activated Gcn2 phosphorylates the eukaryotic initiation factor 2 α-subunit (eIF2α) on Ser 51, which attenuates global translation and triggers the Gcn4-mediated amino acid starvation response (37). Recent work also suggests that ribosome collision may activate the ISR without notable accumulation of uncharged tRNAs (38, 39). Chronic activation of the ISR is implicated in numerous diseases (31). Recent studies suggest that dominant CMT mutations in glycyl-tRNA synthetase (GlyRS) activate the ISR by inducing ribosome stalling (40, 41, 42), and inhibiting the ISR alleviates peripheral neuropathy in a mouse model (40). A pathogenic mutation in glutamyl-tRNA synthetase is also found to impair tRNA charging and activate the ISR (43).

Mutations in the editing sites of AlaRS and ThrRS cause microcephaly and developmental disorders (14, 15, 16). Both AlaRS and ThrRS mischarge Ser onto tRNAs and require editing to remove the mischarged tRNAs. In our previous work, we show that an AlaRS editing-site mutation (C719A) leads to increased Ser mistranslation and activation of the ISR (27), but the underlying mechanism is unclear. Here, we show that Ser misincorporation at Ala and Thr codons induces phosphorylation of eIF2α and *GCN4* expression, which are hallmarks of ISR activation. We further show that activation of the ISR by Ser mistranslation does not require an increase in uncharged tRNAs but rather depends on the P stalk on the ribosome, implicating the involvement of ribosome stalling and collision in mistranslation-induced ISR.

## Results

### Transcriptome analyses of a ThrRS editing–defective yeast strain

**Figure 1:**
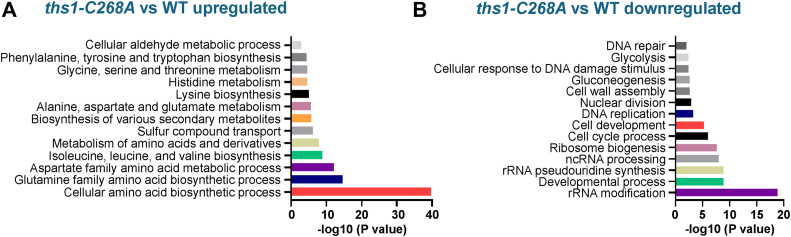
**Transcriptome analysis of ThrRS editing–defective mutant *ths1-C268A* and WT yeast under heat stress.** Cells were grown in YPD to the log phase at 30 °C and shifted to 37 °C for 2 h. Upregulated (*A*) and downregulated (*B*) pathways in the *ths1-C268A* strain are shown. Three biological replicates were performed for each strain. ThrRS, threonyl-tRNA synthetase; YPD, yeast peptone dextrose.

Editing in ThrRS is evolutionary conserved and prevents Ser from being incorporated into Thr codons. We have recently shown that a ThrRS editing–defective yeast strain (*ths1-C268A*) is sensitive to heat stress (26). To understand the global transcriptome changes caused by ThrRS editing defects, we performed RNA sequencing of the WT and *ths1-C268A* strains under heat stress. We grew the cells in yeast peptone dextrose (YPD) media to the log phase at 30 °C and shifted the cultures to 37 °C for 2 h before collecting cells for transcriptome analysis. Compared with the WT, multiple pathways were significantly changed in the ThrRS editing–defective strain (Table S1). Notably, the amino acid biosynthesis pathway was the most significantly upregulated (Fig. 1*A*). In contrast, significantly downregulated pathways include rRNA modification, ribosome biogenesis, cell cycle, DNA replication and repair, tricarboxylic acid cycle, and response to DNA damage (Fig. 1*B*).

### ThrRS editing deficiency activates the ISR in a Gcn2-dependent manner

**Figure 2:**
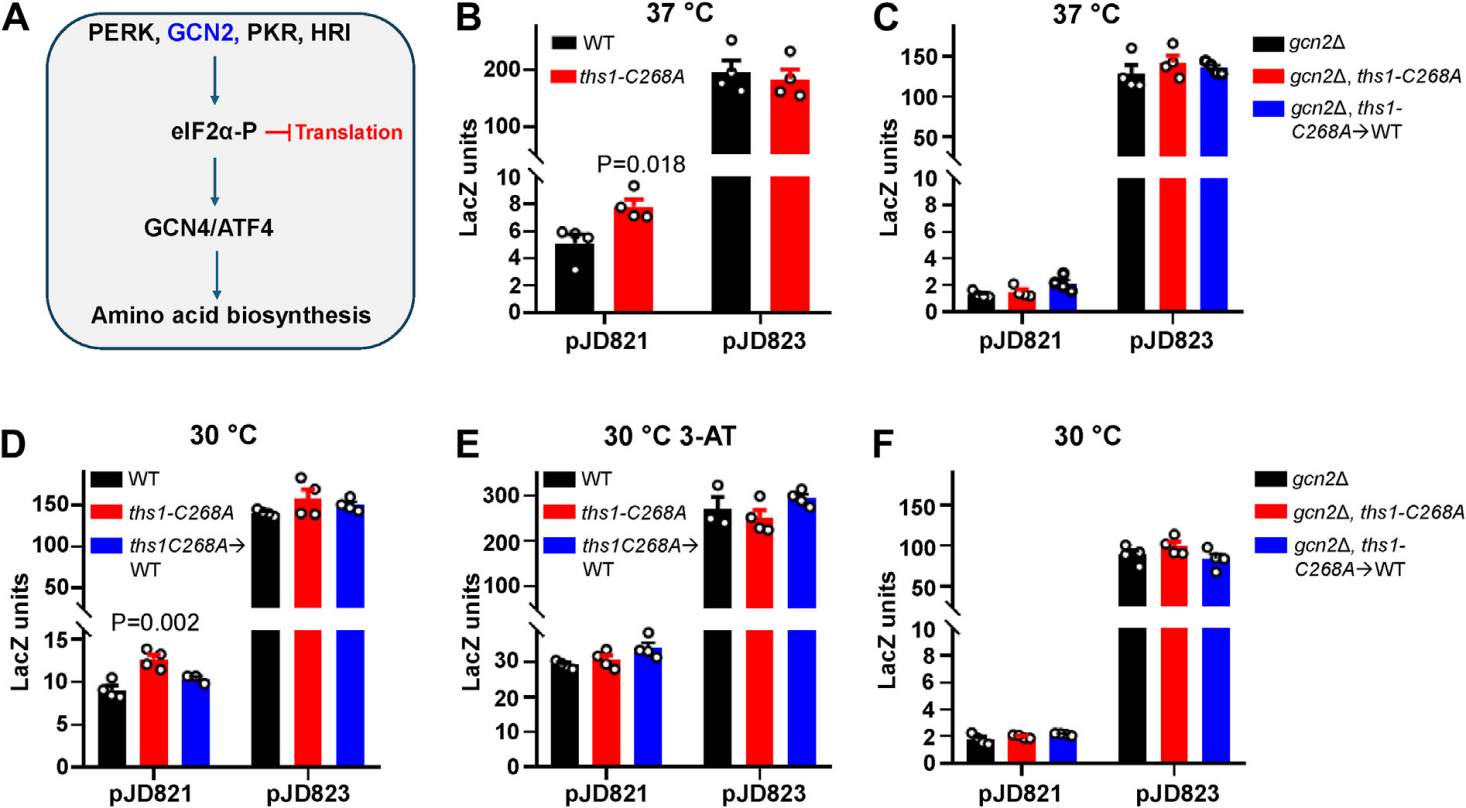
***ths1-C268A* mutation activates the integrated stress response (ISR).***A*, the ISR is activated by four protein kinases, PERK, GCN2, PKR, and HR, in mammals. GCN2 is the only kinase known to activate the ISR in yeast. The ISR attenuates global protein synthesis and activates amino acid biosynthesis *via* GCN4–ATF4. *B*–*F*, expression of *GCN4* shown by LacZ reporters. pJD821 contains all the regulatory elements (uORFs) for *GCN4* expression; pJD823 is a positive control without the regulatory elements. Cells were treated with 100 mM 3-AT before the LacZ assay in (*E*). *GCN4* expression (pJD821) is increased in *ths1-C268A* compared with the WT, suggesting that this mutation activates the ISR at both 30 and 37 °C. Each *circle* represents one biological replicate. Error bars represent one standard deviation from the mean. The *p* values are determined using the unpaired *t* test (*B*) and one-way ANOVA with Dunnett’s test (*C*–*F*). 3-AT, 3-aminotriazole; uORF, upstream ORF.

In yeast, the amino acid biosynthesis pathway is activated by Gcn4 *via* phosphorylation of eIF2α (Fig. 2*A*), which is a hallmark for the activation of the ISR (34, 37). The 5′ of the *GCN4* gene contains four upstream ORFs (uORFs) (34). When the ISR is not activated, translation of these uORFs blocks translation of the downstream *GCN4* ORF. Phosphorylation of eIF2α allows the ribosome to bypass the uORFs and translate *GCN4*. As a reporter for eIF2α phosphorylation and ISR activation, the pJD821 plasmid contains a *lacZ* gene with upstream *GNC4* uORFs (27). As a positive control, pJD823 contains *lacZ* but not uORFs, allowing constitutive expression of *LacZ* independent of ISR activation. We transformed pJD821 and pJD823 into WT and *ths1-C268A* strains. Cells were cultured at 30 °C to the log phase, followed by 2 h of treatment at 37 °C before the β-galactosidase assay. As shown in Figure 2*B*, *GCN4* expression was significantly increased by the *ths1-C268A* mutation, consistent with our RNA sequencing result. In yeast, Gcn2 is the only known kinase to phosphorylate eIF2α. We confirmed that activation of the ISR in *ths1-C268A* was dependent on Gcn2, as deleting the *gcn2* gene abolished *GCN4* expression in the *ths1-C268A* strain (Fig. 2*C*).

**Figure 3:**
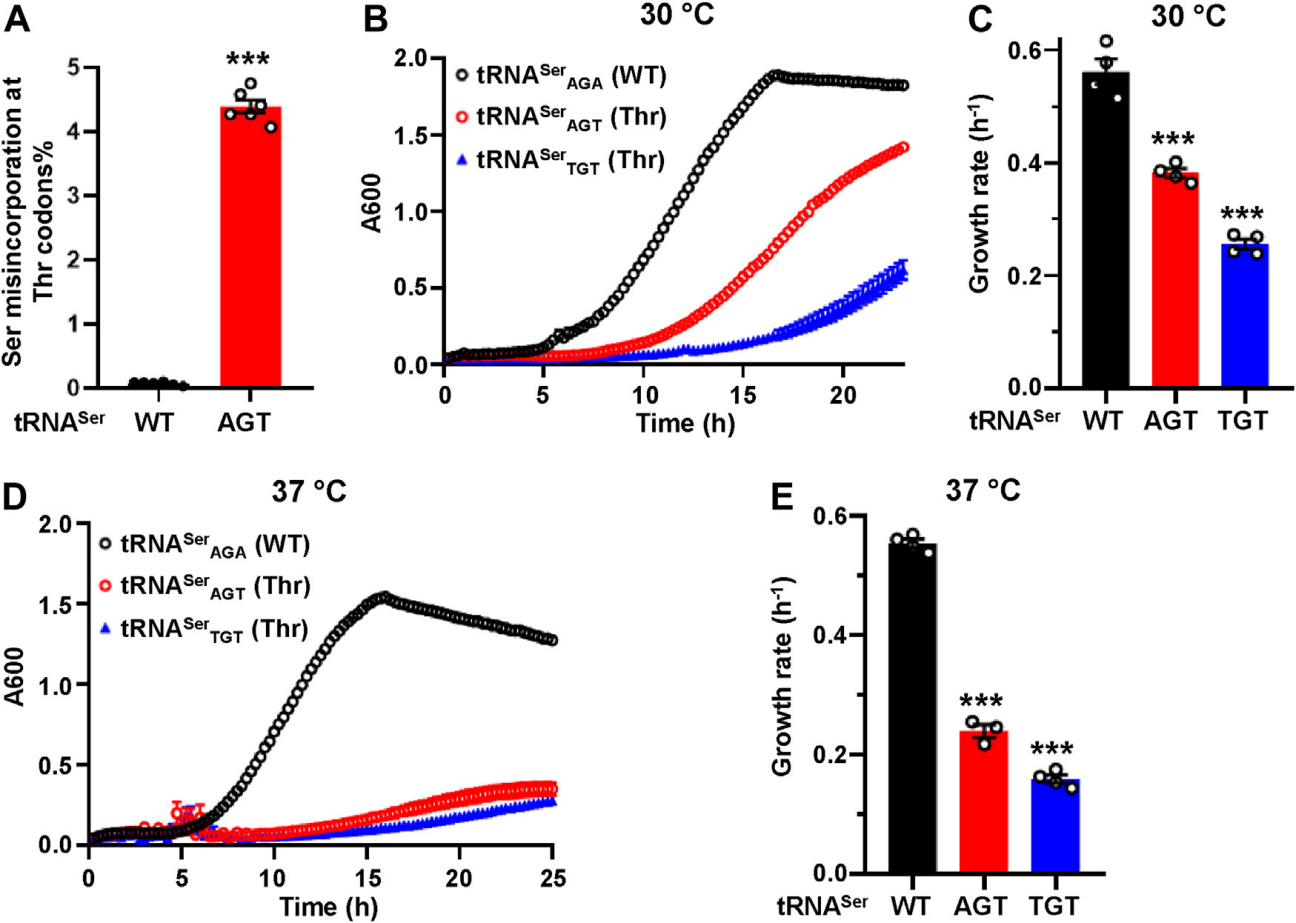
**Serine mistranslation at Thr codons caused by expressing tRNA^Ser^ variants impair growth.***A*, Ser misincorporation at Thr codon was determined using an S68T β-lactamase variant. Growth curves and rates of WT yeast carrying pRS315-tRNA^Ser^ variants at 30 °C (*B* and *C*) and 37 °C (*D* and *E*). *B* and *D*, show the mean growth of at least three biological replicates with error bars indicating one standard deviation. Each *circle* in (*A*, *C*, *E*) represents one biological replicate. *B*, error bars represent one standard deviation from the mean. The *p* values are determined using unpaired *t* test (*A*) and one-way ANOVA with Dunnett’s test (*C* and *E*). ∗∗∗*p* < 0.001.

In addition to abolishing the editing activity, the *ths1-C268A* mutation also decreases the stability of ThrRS at 37 °C (26). A decreased level of aaRSs may lead to the accumulation of uncharged tRNAs and activation of the ISR. Because the stability of the ThrRS C268A mutant protein is not affected at the normal temperature for yeast (30 °C), we decided to test whether an editing defect alone is sufficient to induce the ISR at 30 °C. We found that the *ths1-C268A* mutation also activated the Gcn2-dependent ISR at 30 °C (Fig. 2*D*). We further used the *ths1-C268A*→WT revertant to show that activation of the ISR was indeed because of the *ths1-C268A* mutation (Fig. 2*D*) but not off-target mutations in the CRISPR-engineered strain. Further addition of the His analog 3-aminotriazole increased *GCN4* expression in all three strains to the same level (Fig. 3*E*), and deleting *gcn2* abolished *GCN4* expression at 30 °C (Fig. 3*F*). Collectively, these results support that ThrRS editing deficiency, like AlaRS, activates the ISR in a Gcn2-dependent manner.

### Severe Ser misincorporation at Thr codons by tRNA^Ser^ variants causes growth defects

In addition to aaRS-editing defects, amino acid mistranslation can also result from tRNA variants, particularly in the anticodons. Anticodon variants of tRNAs are indeed frequently found in human populations (44), and mistranslating tRNAs are actively pursued as novel gene therapies to treat genetic diseases (45, 46). To test whether mistranslating tRNAs affect fitness and activate the ISR, we mutated the anticodon of tRNA^Ser^ from AGA (WT) to AGT and TGT to recognize Thr codons instead of Ser codons and expressed the tRNAs in WT yeast on a single-copy plasmid (pRS315). Seryl-tRNA synthetase does not recognize the anticodon and attaches Ser to tRNA^Ser^ anticodon variants (47). Using a β-lactamase reporter assay (26), we found that expressing tRNA^Ser^_AGT_ increased the Ser misincorporation rate at the ACT Thr codon to ∼4% (Fig. 3*A*). Expressing tRNA^Ser^_AGT_ or tRNA^Ser^_TGT_ impaired growth at 30 °C and almost abolished growth at 37 °C (Figs. 3 and S1), suggesting that severe Ser mistranslation is harmful to cells even at normal temperatures. The growth defect at 30 °C is more severe in the tRNA^Ser^_TGT_ strain compared with tRNA^Ser^_AGT_, indicating that Ser mistranslation at ACA/ACG codons is more harmful than at ACT codons.

### Ser misincorporation activates the ISR without increasing uncharged tRNAs

**Figure 4:**
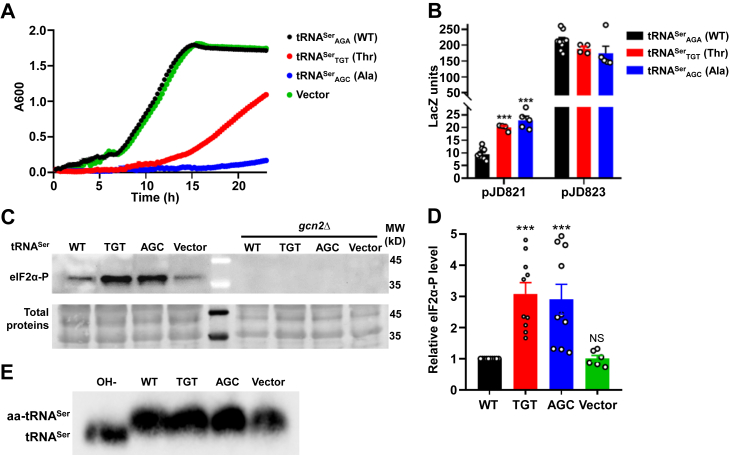
**Mistranslating tRNA^Ser^ variants activates the ISR without increasing uncharged tRNA^Ser^**. *A*, growth curve of WT yeast expressing tRNA^Ser^ variants in SD-Leu medium at 30 °C. The growth curves are the mean of at least three biological replicates with error bars indicating one standard deviation. *B*, expression of *GCN4* shown by LacZ reporters in yeast expressing tRNA^Ser^ variants. *C*, eIF2α phosphorylation detected by Western blot. Total proteins are revealed by Ponceau staining of the transferred membranes. *D*, quantitation of the eIF2α phosphorylation level in the Western blots normalized by total proteins. *E*, acidic northern blot showing aminoacylated and deacylated tRNA^Ser^. OH- treatment deacylates the aa-tRNA. Each *circle* in (*B* and *D*) represents one biological replicate. Error bars represent one standard deviation from the mean. The *p* values are determined using one-way ANOVA with Dunnett's test. ∗∗∗*p* < 0.001. *C* and *E*, show representative images of at least three biological replicates. aa-tRNA, aminoacyl-tRNA; eIF2α, eukaryotic initiation factor 2 α-subunit; ISR, integrated stress response; SD, synthetic defined.

Our results using AlaRS and ThrRS editing–defective strains imply that Ser mistranslation activates the ISR. To validate this concept, we tested whether tRNA^Ser^ mistranslating variants activate the ISR. We transformed plasmids expressing tRNA^Ser^_AGA_ (WT), tRNA^Ser^_TGT_ (Thr), tRNA^Ser^_AGC_ (Ala), and the empty vector. Expressing the WT tRNA^Ser^_AGA_ did not affect growth compared with the vector control but expressing the mistranslating tRNAs decreased growth (Figs. 4*A* and S2). Both tRNA^Ser^_TGT_ and tRNA^Ser^_AGC_ enhanced *GCN4* expression (Fig. 4*B*). *GCN4* expression is controlled by phosphorylation of eIF2α at Ser51 (34). We therefore directly tested eIF2α phosphorylation in the aforementioned yeast strains. As shown in Figure 4, *C* and *D*, expressing tRNA^Ser^_AGA_ (WT) led to a similar base level of eIF2α-P, whereas mistranslating tRNA^Ser^_TGT_ (Thr) and tRNA^Ser^_AGC_ (Ala) significantly increased the level of eIF2α-P, consistent with the *GCN4* reporter results (Fig. 4*B*). Deleting *gcn2* abolished the phosphorylation of eIF2α in all tested strains (Fig. 4*C*), confirming that mistranslation-activated ISR depends on Gcn2.

A possible mechanism of mistranslation-induced ISR is the accumulation of uncharged tRNAs. To test this, we used an acidic northern blot assay to probe the charging level of tRNA^Ser^. We found that tRNA^Ser^ was close to 100% aminoacylated in all four strains (Fig. 4*D*), indicating that Ser mistranslation promotes the ISR without elevating the level of uncharged tRNAs.

### Ser misincorporation activates the ISR *via* the P stalk

**Figure 5:**
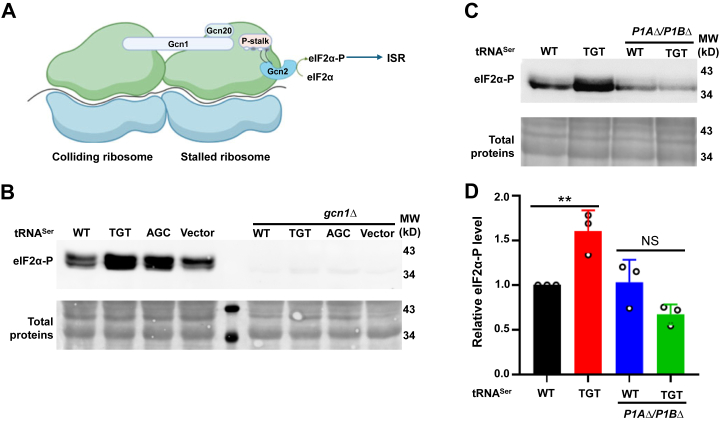
**Activation of eIF2α by Ser mistranslation requires Gcn1 and the P stalk.***A*, ribosome collision is sensed by Gnc1, which recruits Gcn2 to the stalled ribosome. Ribosome stalling caused by inhibitors activates Gcn2 to phosphorylate eIF2α and induce the ISR. *B* and *C*, eIF2α phosphorylation detected by Western blot. Total proteins are revealed by Ponceau staining of the transferred membranes. *D*, quantitation of Western blot results in *C*. Images are representative of at least three biological replicates. eIF2α, eukaryotic initiation factor 2 α-subunit; ISR, integrated stress response.

Our *GCN4* reporter and eIF2α-P results demonstrate that Ser misincorporation activates the ISR through Gcn2 (Figs. 2 and 4). Gcn2 is recruited to the ribosome by Gcn1 upon ribosome collision, and Gcn1 is essential for the association of Gcn2 to the ribosome (39, 48, 49) (Fig. 5*A*). We found that knocking out *gcn1* abolished eIF2α phosphorylation (Fig. 5*B*), indicating that eIF2α phosphorylation (therefore activation of the ISR) upon Ser mistranslation requires Gcn2 to associate with the ribosome. A recent study shows that the P1/P2 stalk on the ribosome is essential for starvation-independent activation (*e.g.*, by ribosome inhibitors) of the ISR (39). Interestingly, accumulation of uncharged tRNAs resulting from starvation does not require the P stalk to activate phosphorylation of eIF2α. We thus tested the role of the ribosome P stalk in mistranslation-induced eIF2α phosphorylation. Deleting both copies of P1 (P1A and P1B) eliminated the difference between the tRNA^Ser^_AGA_ (WT) and tRNA^Ser^_TGT_ (Thr) strains in eIF2α phosphorylation (Fig. 5, *C* and *D*), further supporting that ribosome is needed for eIF2α phosphorylation upon Ser mistranslation. This result also suggests Ser mistranslation activates the ISR through ribosome collision instead of uncharged tRNAs, which is consistent with our acidic northern blot result showing that mistranslating tRNAs do not increase the level of uncharged tRNAs (Fig. 4*D*).

## Discussion

The central dogma explains the flow of genetic information from DNA through mRNA to proteins. Besides aminoacylation, around half of the aaRSs have evolved editing activities to ensure accurate protein synthesis (4, 50). Given the central role of aaRSs in robust and faithful translation, it is not surprising that pathogenic mutations in aaRSs are increasingly associated with various human diseases, including peripheral neuropathies (8, 9, 51), brain development (52, 53), neurodegenerative diseases like Alzheimer’s disease (54), autoimmune disorders (55), cancer (18, 56), and cardiovascular diseases (57, 58, 59, 60). Almost all cytosolic and mitochondrial aaRSs are implicated in the pathology of the human nervous system (53, 61). CMT, a peripheral neurological disease characterized by muscle weakness and limb atrophy, was the first discovered neurological disorder to be linked to aaRS mutations (8). Cytosolic aaRS mutations leading to CMT are dominant and do not often cause aminoacylation defects (9). Recent studies using mice models and human cell lines suggest that chronic activation of the ISR plays a central role in CMT caused by aaRS mutations (40). CMT-causing GlyRS (or GARS) mutants sequester tRNA^Gly^ and reduce the supply of Gly-tRNA^Gly^ to the ribosome, leading to ribosome stalling and activation of the ISR. Inhibiting ISR by deleting *gcn2* or using a small-molecule inhibitor ISRIB remarkably alleviates the symptoms in mice (40). Overexpressing tRNA^Gly^ in GlyRS mutant flies and mice also suppresses the ISR and rescues the neuropathy phenotype (41). Whether ISR activation is responsible for peripheral neuropathies caused by other aaRS mutations remains to be determined.

In contrast to the dominant aaRS mutations affecting peripheral neurons, cytosolic aaRS mutations affecting the central nervous system are biallelic and recessive (9, 52). Biallelic aaRS mutations identified in patients often decrease the aminoacylation efficiency or the stability of the mutant aaRSs (14, 15, 16, 52). Decreased aminoacylation efficiency has been shown to activate the ISR in yeast and mammalian cells (27, 43), likely through the accumulation of uncharged tRNAs. Multiple pathogenic mutations have also been mapped to the editing sites of AlaRS and ThrRS (14, 15, 16). Here, we show that Ser misincorporation at Ala and Thr codons robustly activates the ISR. Interestingly, the tRNA charging level appears to be unchanged (Fig. 4*D*), raising the question as to how mistranslation activates the ISR. Recent studies reveal that ribosome stalling and collision can induce the ISR (38, 39, 62, 63). Recruitment of Gcn2 to the ribosome depends on Gcn1 (48, 49), which binds to stalled ribosomes as revealed by structural analyses (64). We show that deleting *gcn1* or the P stalk of the ribosome abolishes phosphorylation of eIF2α in mistranslating strains (Fig. 5), leading us to speculate that Ser mistranslation results in aberrant translation elongation that leads to ribosome stalling. How this occurs remains an intriguing question for future exploration. It is important to note that not all mistranslation events activate the ISR. Indeed, previous work shows that editing defects in PheRS attenuate, rather than activate, the ISR in yeast (65). One possible explanation for the difference between various mistranslation events is that misincorporation of Ser at Ala and Thr codons specifically leads to defective ribosomes with slow kinetics. Alternatively, Ser misincorporation may directly slow down ribosome elongation at specific codons. Our work thus underscores the heterogeneous cellular responses to different types of translational errors.

## Experimental procedures

### Materials, media, and strains

All *Saccharomyces cerevisiae* strains used here were derivatives of BY4741. *Escherichia coli* DH5α grown in LB medium was used for molecular cloning. Gene knockout was generated by replacing the coding regions with the *HIS2* or *LEU* gene and verified by PCR. Yeast point mutation mutants and gene knockout strains were grown in the YPD media (1% yeast extract, 2% peptone, and 2% glucose). To induce Ser misincorporation into Thr or Ala codons, the anticodon of tRNA^Ser^ was mutated and cloned to pRS315. For strains harboring plasmids, they were grown in synthetic defined (SD) dropout medium (0.17% yeast nitrogen base, 0.5% ammonium sulfate, 2% glucose, and 0.14% amino acid dropout mix, -His, -Leu, or -Ura).

### Transcriptome analysis

WT and *ths1-C268A* cells were grown at 30 °C to the midlog phase and further cultured at 37 °C for 2 h. Total RNA was prepared using an RNA extraction kit (Qiagen). Library construction and Illumina sequencing were performed by Novogene.

### Spot assay

Yeast cells from single colonies were resuspended in SD-Leu, grown at 30 °C to saturation, and diluted 1:50 for continued growth to the log phase. Aliquots with serial dilutions (10^0^ to 10^−5^) were spotted on SD-Leu agar plates, which were incubated at 30 °C or 37 °C for 3 days before imaging.

### Growth and temperature sensitivity analysis

The yeast cells were grown in YPD or SD-Leu at 30 °C to saturation and 1:50 in YPD or SD-Leu in 96-well plate. Growth was performed in a microplate reader (Synergy H1; BioTek).

### **β**-lactamase assay

A β-lactamase assay was used to determine the Ser misincorporation rate in the yeast cells as described (26, 27).

## Data availability

The RNA-sequencing data are deposited in Genome Expression Omnibus (accession no.: GSE284986): https://www.ncbi.nlm.nih.gov/geo/query/acc.cgi?acc=GSE284986.

## Supporting information

This article contains supporting information (26).

## Conflict of interest

The authors declare that they have no conflicts of interest with the contents of this article.
