# Supplementary material for: Serine mistranslation induces the integrated stress response through the P stalk
Source: J Biol Chem. 2025 Mar 25;301(5):108447. doi: 10.1016/j.jbc.2025.108447 (PMC12022490; doi:10.1016/j.jbc.2025.108447)
Supplement: Supporting information [file mmc1.docx]

SUPPORTING INFORMATION

**Serine mistranslation induces the integrated stress response through the P stalk**

Hong Zhang^1*^, Jiqiang Ling^1*^

^1^Department of Cell Biology and Molecular Genetics, The University of Maryland, College Park, MD 20742, USA

*Correspondence should be addressed to:

Hong Zhang: +1 (301) 405-1035; Email: hzhang21@umd.edu.

Jiqiang Ling: +1 (301) 405-1035; Email: [jling12@umd.edu](mailto:jling12@umd.edu).

**Supplemental experimental procedures**

***β-galactosidase assay***

To test the expression of *GCN4*, yeast strains carrying pJD821 and pJD823 reporters were grown in SD dropout media to A600 of 1.0-1.2 at 30 °C. 700 µl of cultures were collected and resuspended in 700 µl Z-buffer (60 mM Na_2_HPO_4_, 40 mM NaH_2_PO_4_, 10 mM KCl, and 1 mM MgSO_4_), and cell density was measured by OD_600_. Cells were lysed by adding 100 µl Chloroform and 50 µl 0.1% SDS and vortexing for 15 s. The reaction was started by the addition of 0.2 ml prewarmed ONPG (o-nitrophenyl-β-galactoside, 4 mg/ml in Z-buffer), and terminated by adding 500 µl Na_2_CO_3_ (1 M). Cell debris was removed by centrifugation, and OD_420_ of the supernatant was determined using a platereader (Synergy HT, BioTek). The β-galactosidase activity was calculated according to the following equation: LacZ units (or Miller units) = 1000*OD_420_/(time*volume*OD_600_).

***Acidic northern blot***

Total RNA of yeast cells was extracted using a hot phenol method. Pellets were dissolved in sodium acetate buffer (pH5.2). All samples were stored at -80 °C till use. Acidic northern blot was conducted as described (1).

***Western blot***

Western blot was essentially performed as described (1). Yeast cultures were grown 10-30 h to A600 of 1.0-1.2, and total proteins were extracted using TCA precipitation. Samples were separated on 12% SDS-PAGE gels and transferred to nitrocellulose membranes, A standard Western blotting procedure was followed. The rabbit anti-eIF2α-P antibody (Thermo) with 1:1000 dilution and goat anti-mouse IgG-HRP secondary antibody (Invitrogen) with 1:5000 dilution were used. The anti-eIF2α-P antibody was validated with the *gnc2*Δ strain and the correct molecular weight. Ponceau S Staining was used to reveal the total proteins according to the manufacturer’s protocol (Acros Organics). Nitrocellulose membranes were treated with ECL chemiluminescent substrate reagents (Bio-Rad) and visualized using a ChemiDoc Imaging System (Bio-Rad). The signals of eIF2α-P were quantitated using Image J (National Institutes of Health, USA) and normalized with total proteins.


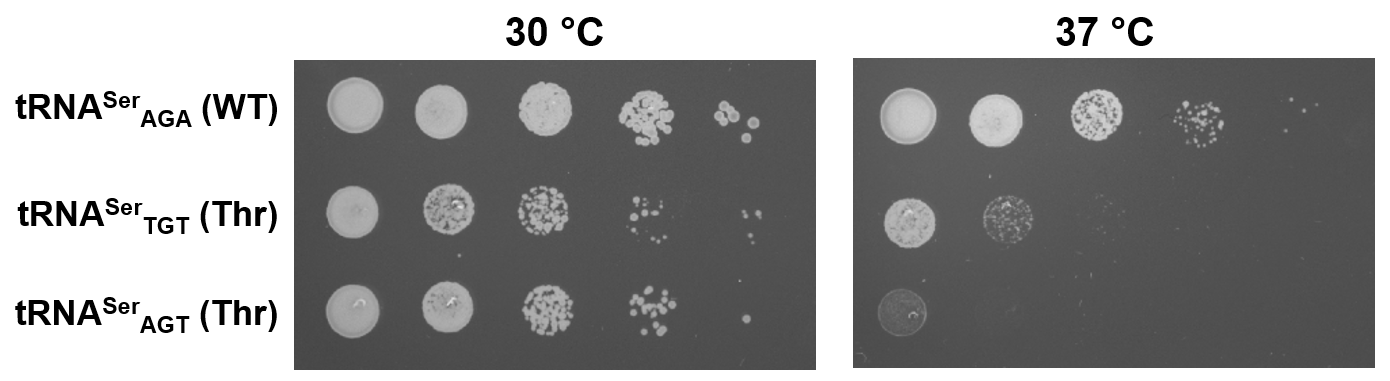


**Figure S1. Growth of yeast strains expressing tRNA^Ser^ variants.** 10-fold serial dilutions of saturated liquid cultures were performed, and aliquots were sported on agar SD-Leu agar plates, which were incubated at 30 or 37 °C for 3 days before imaging. The images are representatives of four biological replicates.


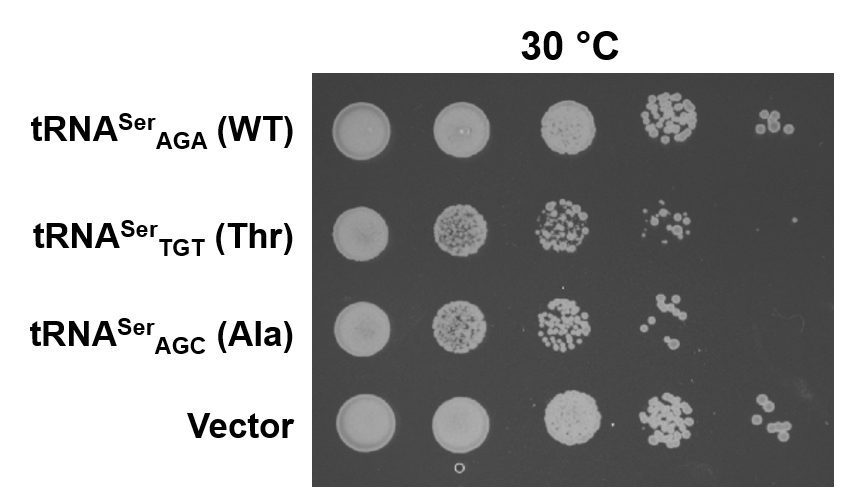


**Figure S2. Growth of yeast strains expressing tRNA^Ser^ variants and a vector control.** The assay was performed as in Figure S1.

1. Zhang, H., Murphy, P., Yu, J., Lee, S., Tsai, F. T., van Hoof, A., and Ling, J. (2023) Coordination between aminoacylation and editing to protect against proteotoxicity. *Nucleic Acids Res* **51**, 10606-10618
